# Stress Fracture of the Trapezoid in a Professional Tennis Player

**DOI:** 10.31486/toj.23.0067

**Published:** 2024

**Authors:** Jimmy C. Daher, Esther Tannoury, Joey Daher, Stephanie Chahwan, Sahar Semaan

**Affiliations:** ^1^ Department of Orthopedic Surgery, Lebanese American University Medical Center-Rizk Hospital, Beirut, Lebanon; ^2^ Department of Orthopedic Surgery, Ochsner Clinic Foundation, New Orleans, LA; ^3^ Department of Radiology, Lebanese American University Medical Center-Rizk Hospital, Beirut, Lebanon; ^4^ Department of Family Medicine, Lebanese American University Medical Center-Rizk Hospital, Beirut, Lebanon

**Keywords:** *Carpal bones*, *fractures–stress*, *metacarpal bones*, *racket sports*, *trapezoid bone*

## Abstract

**Background:** Repetitive microtrauma can lead to trapezoid and second metacarpal stress fractures in racket sport players. Nontraumatic trapezoid stress fractures are rare and difficult to diagnose. To our knowledge, only 3 cases had been reported as of May 2023. We report the fourth case of a nontraumatic sports-related trapezoid stress fracture and only the second case in a tennis player.

**Case Report:** A 29-year-old professional and right hand–dominant male tennis player presented with right hand and wrist pain for 3 weeks. He complained of dorsal wrist tenderness proximal to the base of the second metacarpal that was exacerbated by extension of the index finger. Initial radiographs were normal, but magnetic resonance imaging of the wrist showed a stress fracture of the trapezoid bone and base of the second metacarpal. The patient was treated conservatively with a wrist brace, cessation of sports activities, and modification of his training routine. The patient was asymptomatic at 1-year follow-up.

**Conclusion:** This case highlights the relationship between trapezoid and second metacarpal stress fractures in athletes. A high index of suspicion for trapezoid stress fractures should be maintained and included in every differential diagnosis for athletes, especially racket sport players presenting with wrist pain. To avoid future injuries, clinicians should not only treat the fracture but also address the risk factors predisposing to this injury.

## INTRODUCTION

Trapezoid fracture is the least common carpal bone fracture, accounting for only 0.4% of total carpal bone fractures.^1^ The rarity of this fracture is because of the shape and position of the trapezoid within the wrist joint. The trapezoid is tightly located between several other carpal bones and the base of the second metacarpal and is firmly attached to them through strong intercarpal ligaments, thus making the bone relatively immobile.^1-3^ The trapezoid has a wedge shape and is 2 times wider dorsally than it is volarly, making it a keystone for the carpal arch.^1^ The mechanism of injury described in the literature usually involves a high-energy trauma or fall. In these situations, a considerable amount of axial force or bending stress is transmitted indirectly from the second metacarpal base to the trapezoid with or without wrist flexion or extension.^3,4^ Such traumatic injuries are usually accompanied by concomitant fractures of other carpal bones and metacarpals.^4,5^ On the other hand, nontraumatic trapezoid fractures are extremely rare, with, to our knowledge, only 3 cases reported as of May 2023. All 3 cases were diagnosed as stress fractures and were related to sports: tennis, shot put, and baseball.^4-6^ We report the fourth case of nontraumatic sports-related trapezoid stress fracture and the second case in a tennis player.

Because our patient also had a stress fracture of the base of the second metacarpal, we conducted a literature review of nontraumatic trapezoid and second metacarpal stress fractures with a focus on the characteristics of such fractures in racket sport athletes.

## CASE REPORT

**Figure 1.**
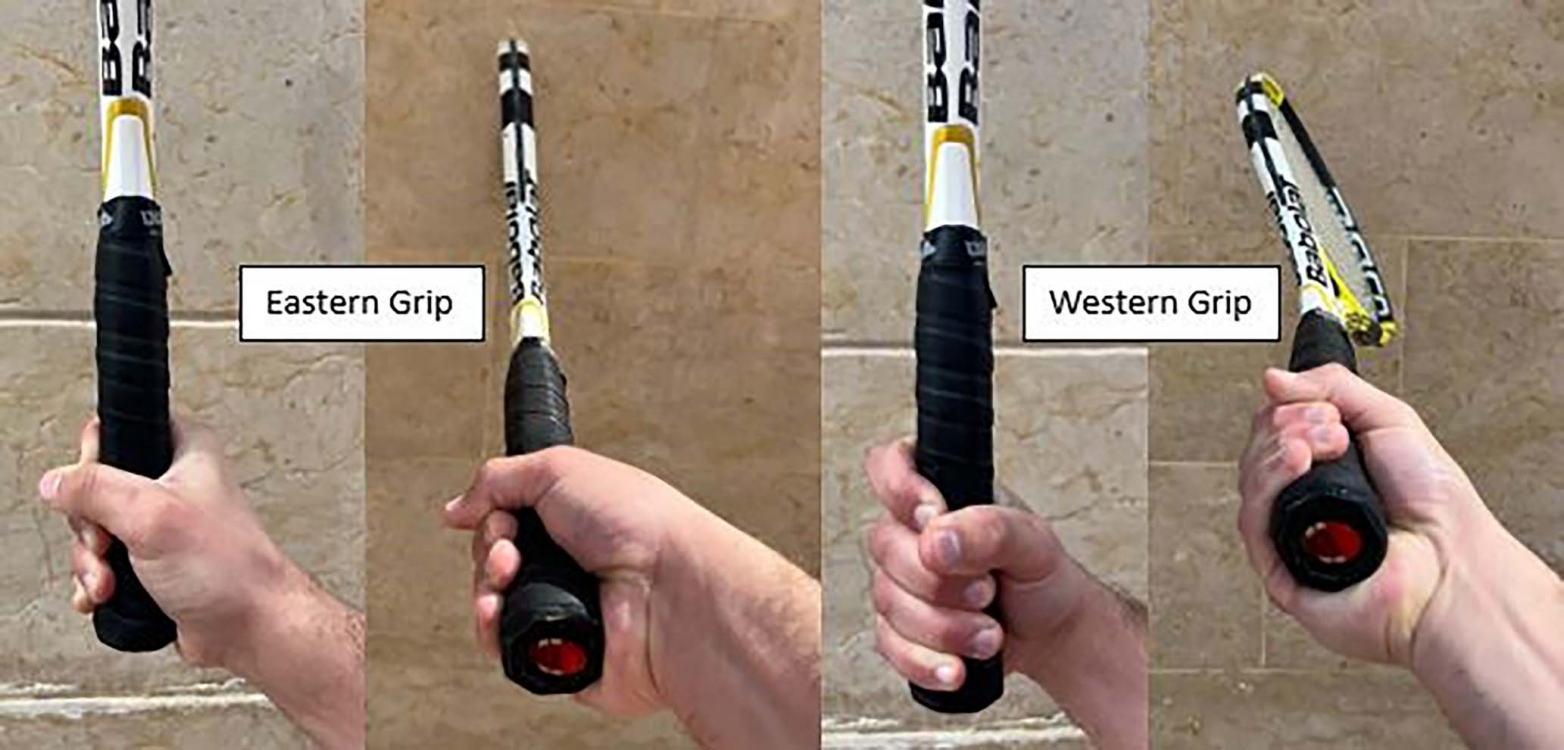
Eastern and western grip techniques.

**Figure 2.**
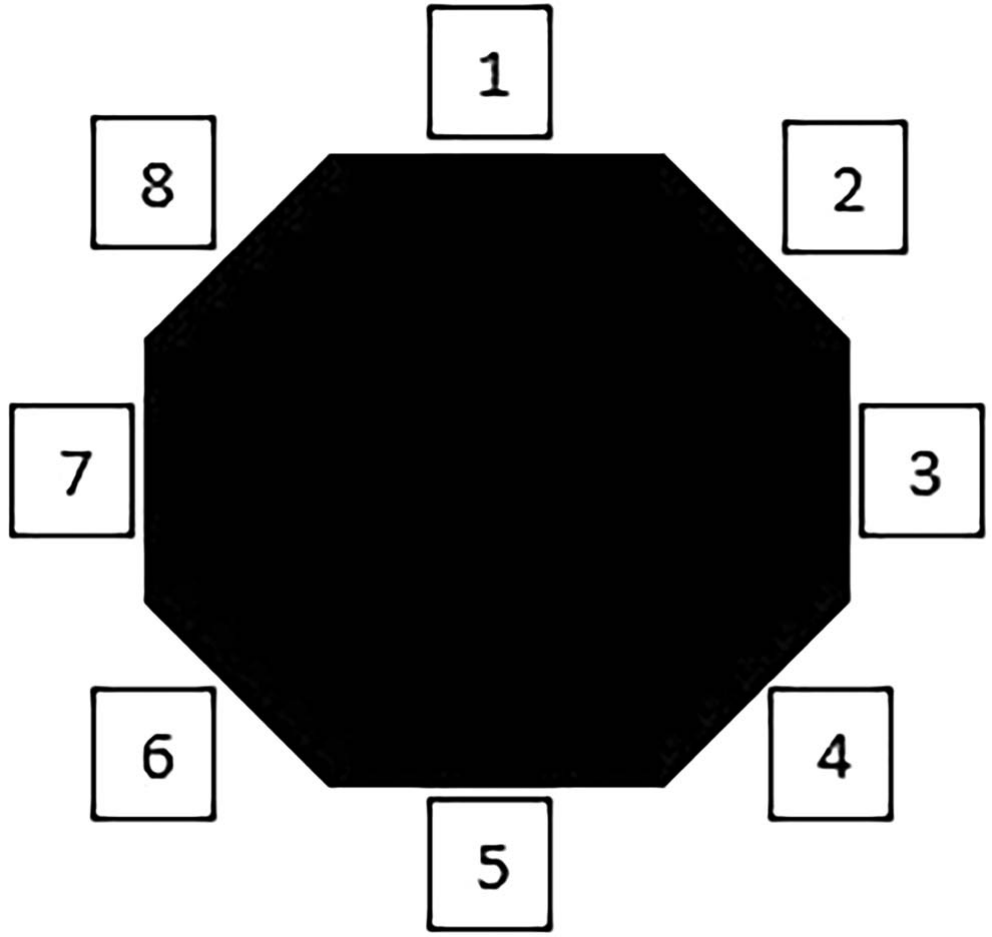
Racket handle bevel numbering for a right hander. Bevel 3: location of the palm side of the index finger knuckle in an eastern grip technique; Bevel 5: location of the palm side of the index finger knuckle in a western grip technique.

A 29-year-old professional right hand–dominant male tennis player presented to our facility complaining of right dorsal hand and wrist pain for the prior 3 weeks. The pain was localized at the region of the base of the second metacarpal and had progressively increased when the patient doubled the amount and intensity of his tennis training to 3 hours daily in preparation for a major tournament. The patient denied any history of trauma. He swung his racket using the western grip technique (Figures 1 and 2) and experienced pain principally during serving and forehand strokes.

**Figure 3.**
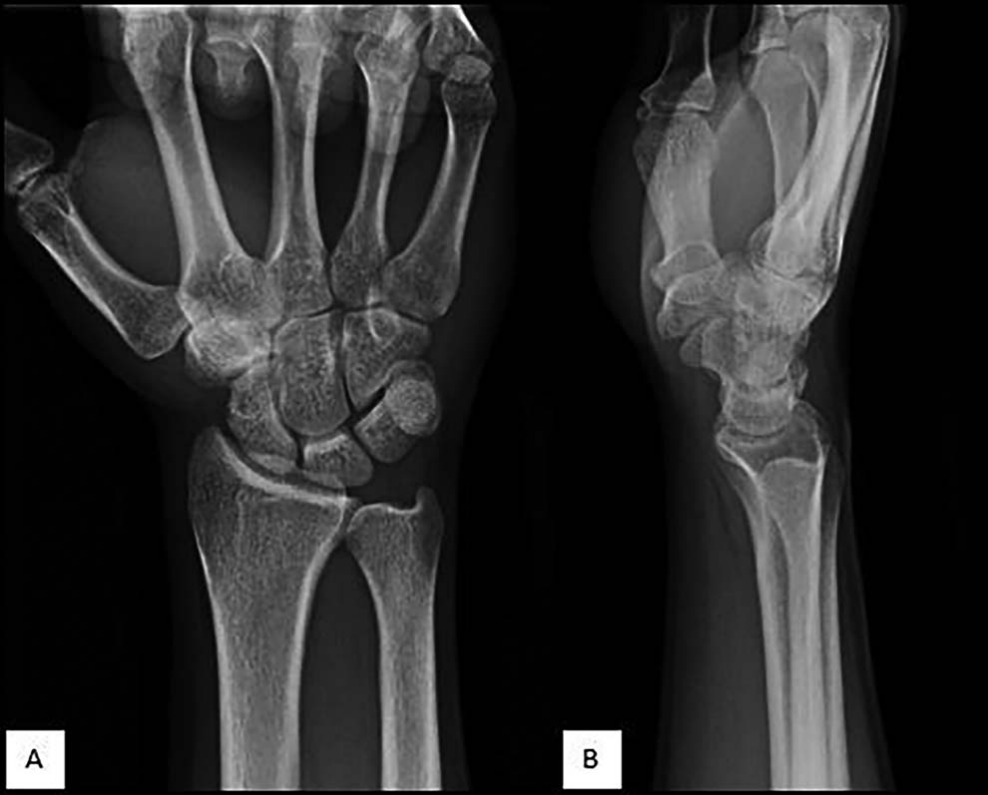
Unremarkable (A) posteroanterior and (B) lateral radiographs of the right wrist show no evidence of acute fracture.

On physical examination, the patient had point tenderness over the dorsal aspect of the wrist proximal to the level of the base of the second metacarpal. He complained of pain only during extension of the index finger. Otherwise, he had full nonpainful active range of motion (ROM) of the wrist and fingers and had no swelling, masses, or deformities. Radiographs of the right wrist were normal (Figure 3). Tendonitis of the extensor tendons was suspected, and the patient was treated conservatively with nonsteroidal anti-inflammatory drugs (NSAIDs) without avoidance of sports. However, the pain persisted and worsened after 1 week.

**Figure 4.**
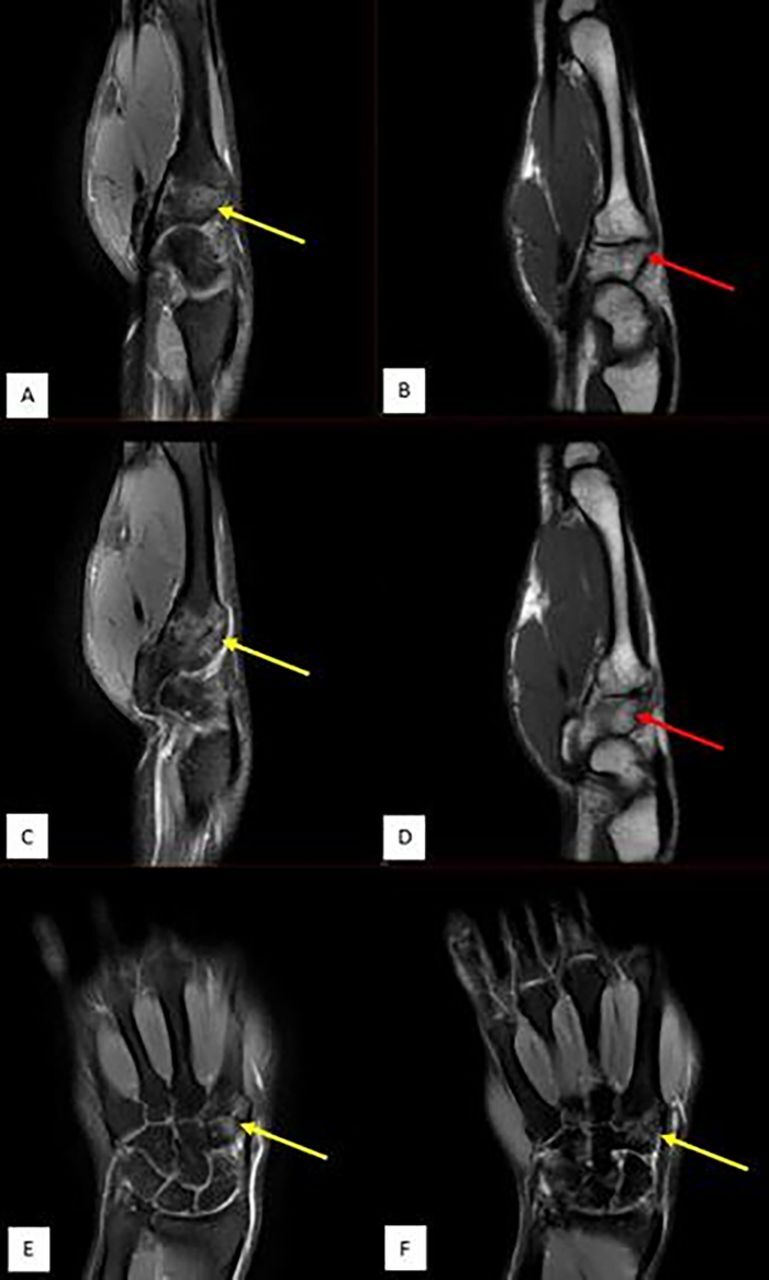
Magnetic resonance imaging of the right wrist—(A and C) sagittal fat suppressed proton density (PD FS), (E and F) coronal PD FS, and (B and D) sagittal T1 images—showing bone marrow edema on opposing sides of the second carpometacarpal joint, predominantly involving the radial aspect of both trapezoid and base of the second metacarpal bones (yellow arrows). Imaging shows no evidence of periosteal reaction or low T1 fracture line (red arrows show only ill-defined edema), and findings are in keeping with a microtrabecular injury.

Magnetic resonance imaging (MRI) showed bone marrow edema of the trapezoid and radial aspect of the base of the second metacarpal (Figure 4). The patient was diagnosed with a stress fracture of both the trapezoid bone and base of the second metacarpal and was treated with a wrist brace for 4 weeks along with avoidance of all sports. The patient was also advised to revert to his initial training routines without doubling the amount and intensity and to change his grip technique if possible. Our patient did not change his grip technique, but he altered his stroke biomechanics and decreased his training intensity. He was symptom-free at 6-week and 1-year follow-ups.

## DISCUSSION

Although metacarpal stress fractures are uncommon and account for only 1.4% of all fractures,^7,8^ a carpal stress fracture percentage has not been reported. Furthermore, trapezoid fractures are even rarer because of the position and shape of the trapezoid in the wrist joint as described previously.^1-3^ Identification of a trapezoid fracture is usually easier when concomitant fractures coexist in the same joint. Such injuries occur as a result of direct high-energy trauma, and imaging such as MRI or computed tomography (CT) scans are usually ordered at presentation for diagnosis.^9,10^ On the other hand, the mechanism of injury for nontraumatic trapezoid stress fractures is repetitive impact loading, principally on the dorsal aspect of the trapezoid bone in the dominant wrist and thus seen primarily in racket sport athletes.^4-6^ A study by Anderson revealed that only 15% of initial radiographs are positive for upper extremity stress fractures, and the incidence increases to only 50% over time.^11^ In addition, because of the overlap of the carpal bones in the wrist joint, a trapezoid fracture can be easily missed with plain radiographs.^2,3,5,12^

Based on the case reports in the literature, clinical presentation seems to slightly differ among patients with traumatic trapezoid fractures. Sadowski and Montilla reported a patient with traumatic trapezoid fracture who presented with swelling, decreased ROM of the wrist, pain at the base of the second metacarpal, and snuffbox tenderness.^12^ Afifi and Lu reported similar symptoms after trauma but with the absence of snuffbox tenderness.^2^ Nammour et al described 2 patients with traumatic trapezoid fractures.^3^ One patient presented with swelling, limited ROM of the wrist, and tenderness at the radial aspect of the wrist, while the other patient complained of dull pain at the snuffbox with scaphoid tenderness but no swelling or restricted ROM.^3^

For nontraumatic sports-related stress fractures, clinical features also varied among the 3 patients reported in the literature and differed from the traumatic cases as well.^4-6^ A baseball player presented with swelling and tenderness over the trapezoid region, pain only with axial compression of the index finger, and normal ROM in the wrist.^6^ The shot putter presented with pain and tenderness at the proximal aspect of the first and second metacarpal that was exacerbated by thumb and index finger extension and abduction.^5^ The tennis player presented with wrist pain and point tenderness over the trapezoid, minimal tenderness over the scapholunate interval, and no wrist swelling or ROM restriction.^4^ Our patient presented with pain and tenderness over the region of the trapezoid that was exacerbated with index finger extension but no swelling or ROM restriction.

With such an unpredictable clinical presentation and the low incidence of fracture detection on plain radiographs at initial presentation, clinicians should have a high index of suspicion for these fractures. Depending on the clinical presentation, further investigation should be carried out either by CT scan, MRI, or bone scintigraphy.^5,6^

The second metacarpal bone is long, has the widest base compared to other metacarpals, and articulates with the trapezoid, trapezium, capitate, and third metacarpal. The base of the second metacarpal bone receives maximum tension when the hand grips a tool.^13^ Simultaneously, slight metacarpal bone flexion can cause an axial load force on the dorsal aspect of the trapezoid, and this force can ultimately cause the trapezoid to fracture or dorsally dislocate.^4,14^ Stirling and Oliver discussed 19 cases of metacarpal stress fractures in sports, and the second metacarpal was the most commonly injured bone (13 cases, 68%).^15^

Because of the rarity of nontraumatic sports-related trapezoid fractures and the close relationship between the trapezoid and second metacarpal, we conducted a literature review targeting both bones and focusing on racket sport athletes with the goal of providing a guide for physicians to diagnose such fractures without delays. We summarize the clinical presentation of each patient and address the risk factors and clinical outcomes after management.

In our literature review, we found 26 sports-related stress fractures, with 3 cases involving the trapezoid and 23 cases involving only the second metacarpal.^4-6,13,14,16-22^ The data are summarized in Table 1. The time from the onset of symptoms to the clinical presentation ranged from as early as 7 days to 2 years, with the majority of patients seeking consultation within 1 month.^4,6,13,14,16-19,21,22^ All patients received initial radiographs, and they were positive in 9 cases (35%).^16-18,20-22^ This percentage of positive radiographs is higher than the 15% described by Anderson^11^; the difference could be explained by the varying time of presentation for each case. Nevertheless, the incidence did not exceed 50%. Initial radiographs for the trapezoid fractures were negative in all 3 patients.^4-6^ Other imaging modalities reported were MRI (the most common at 20 cases), followed by bone scintigraphy (5 cases) and CT scan (3 cases). No consensus has been reached on what type of imaging should be used after an initial radiograph. While bone scintigraphy has been used,^5,6,17,20^ MRI and CT scans are faster, are more readily available, and provide detailed information that can guide treatment.^3^ Blomquist et al showed that MRI has a higher sensitivity and specificity compared to CT scan in identifying trapezoid fractures and provides additional information regarding secondary injuries whether they are soft tissue or bone related.^4^

**Table 1.** Reports of Nontraumatic Stress Fractures of the Trapezoid and Second Metacarpal Bones

Study	Age, years/Sex	Time From Onset of Symptoms to Presentation, days	Stress Fracture Location	Type of Sport	Imaging Modalities for Diagnosis	Treatment Type and Duration	Pain Free After Treatment, weeks
Nagumo et al, 2002^6^	21/M	30	Trapezoid	Baseball (batter)	X-ray: normal Bone scintigraphy: increased uptake in trapezoid bone MRI: stress fracture of trapezoid bone with a dorsal bone fragment	Dorsal surgical approach to remove dorsal fragment and refrain from batting (4 wks)	208 (4 yrs)
Heron et al, 2012^5^	22/NA	365-730 (1-2 yrs)	Trapezoid	Shot put	X-ray: normal Bedside ultrasound: normal Bone scintigraphy: increased uptake in trapezoid bone CT: stress fracture of the trapezoid	Thumb spica splint (8 wks)	N/A
Blomquist et al, 2013^4^	21/F	30	Trapezoid	Tennis	X-ray: normal CT: no definite fracture MRI: marrow edema in the trapezoid with a coronally oriented fracture in the midtrapezoid on the sagittal projection	Avoidance of sport activity, PT, splint immobilization (6 wks)	4
Murakami, 1988^16^	16/M	7	Base of 2nd metacarpal	Tennis	X-ray: periosteal reaction at ulnar aspect of base of 2nd metacarpal	Avoidance of sport activity (4 wks) + change of grip technique	6
Waninger and Lombardo, 1995^17^	14/F	10	Base of 2nd metacarpal	Tennis	X-ray: periosteal reaction at ulnar aspect of base of 2nd metacarpal Bone scintigraphy: positive for stress fracture	Avoidance of sport activity (2 wks) + PT and change to eastern grip technique	6
Bespalchuk et al, 2004^18^	15/F	30	Base and shaft of 2nd metacarpal	Tennis	X-ray: periosteal reaction at base and midshaft of 2nd metacarpal CT: cortical thickening at base and shaft of 2nd metacarpal MRI: stress fracture of base and shaft of 2nd metacarpal	Avoidance of sport activity (4 wks) + change to eastern grip technique	12
Muramatsu and Kuriyama, 2005^14^	13/F	21	Base of 2nd metacarpal	Soft tennis	Initial x-ray: normal (done elsewhere) X-ray at 3 wks: fracture line and periosteal reaction at base of 2nd metacarpal MRI: stress fracture of base of 2nd metacarpal	Avoidance of sport activity (5 wks) + change to eastern grip technique	12
Fukuda et al, 2008^19^	14/F	21	Base of 2nd metacarpal	Badminton	Initial x-ray: normal (done elsewhere) X-ray at 5 wks: periosteal reaction at ulnar aspect of base of 2nd metacarpal	NSAIDs without limiting activity for 5 wks (failed) followed by avoidance of sport activity (3 wks)	5 (after sport restriction)
Balius et al, 2010^20^	17/F	NA	Shaft of 2nd metacarpal	Tennis (all patients)	X-ray: normal (3 patients) X-ray: periosteal reaction and hairline crack in the cortex (3 patients) Bone scintigraphy: positive (performed in 2 patients with positive x-rays) MRI: increased bone marrow signal intensity (all patients)	PT and avoidance of sport activity (6 to 8 wks) for all patients	6
	17/F		2nd metacarpal				7
	15/F		Shaft of 2nd metacarpal				6
	17/M		2nd, 3rd, and 4th metacarpal				10
	15/F		Shaft of 2nd metacarpal				6
	17/F		2nd metacarpal				6
Rolison and Smoot, 2017^21^	19/M	14	Shaft of 2nd metacarpal	Golf	X-ray: periosteal reaction at ulnar aspect of shaft of 2nd metacarpal MRI: marrow edema in the second metacarpal shaft suggesting stress fracture	Wrist splint, avoidance of sport activity (8 wks) + modified grip technique	16
Duarte et al, 2017^13^	27/M	28	Shaft of 2nd metacarpal	Tennis	X-ray: normal MRI: light bone edema in the 2nd metacarpal shaft with periosteal reaction and 2 lines of hyposignal suggesting stress fracture	Cast immobilization (4 wks) + change of grip	8
Nishikawa et al, 2020^22^	13/F	28	Shaft of 2nd metacarpal	Badminton	X-ray: periosteal reaction at ulnar aspect of shaft of 2nd metacarpal (1 patient/tennis player) X-ray: positive for stress reaction (1 patient/location and type of sport NA) X-ray: normal (8 patients) MRI: stress fracture of metacarpal bones (in 8 patients with normal x-rays) MRI: not performed for the 2 patients with abnormal x-rays	All patients treated with avoidance of sports activities + change of grip technique in some patients (advised to change from western to eastern)	4
	14/F	7	Base of 2nd metacarpal	Soft tennis			12
	14/M	21	Base of 2nd metacarpal	Badminton			4
	15/M	28	Base of 2nd metacarpal	Tennis			3
	16/M	14	Shaft of 2nd metacarpal	Tennis			4
	16/F	21	Base of 2nd metacarpal	Tennis			10
	18/F	7	Shaft of 2nd metacarpal	Tennis			4
	18/M	364	Shaft of 2nd metacarpal	Boxing			5
	22/F	7	Shaft of 2nd metacarpal	Tennis			4
	24/M	14	Shaft of 2nd metacarpal	Bowling			4

CT, computed tomography; F, female; M, male; MRI, magnetic resonance imaging; NA, not available; NSAIDs, nonsteroidal anti-inflammatory drugs; PT, physical therapy; x-ray, plain radiograph.

Except for 1 patient who underwent surgery,^6^ all other patients were treated conservatively by either immobilization with splinting/casting or avoiding sports for 4 to 8 weeks.^4,5,13,14,16-22^ Some patients also underwent physical therapy and changed their grip technique, especially in racket sports.^13,14,16-18,21,22^ Symptoms in only 1 patient worsened after her initial visit because she was treated with NSAIDs for 5 weeks without limiting her activity.^19^ The patient ultimately improved after cessation of sports. This result is somewhat similar to our case in which the patient's symptoms worsened after 1 week of treatment of NSAIDs without avoiding sports activities, thus highlighting the importance of sports cessation during the first few weeks of treatment to relieve the repetitive stress on the fractured bone. Our patient was then managed conservatively with a short arm wrist brace and avoidance of sport activities for 4 weeks. Our patient did not change his grip technique, but he decreased the intensity of his training and adjusted his stroke biomechanics. This management was consistent with several studies that support conservative management by limiting sport activities or immobilizing the extremity for nondisplaced fractures and limiting surgical intervention to those with displaced fractures or concomitant fractures.^3,6,13,20,23^

The most common type of sport associated with trapezoid and second metacarpal fractures was racket sports (21/26, 81%), including tennis, badminton, and soft tennis (16 vs 3 vs 2 cases respectively, Table 1). Table 2 summarizes the clinical presentations and sports characteristics of the racket sport athletes who were diagnosed with either a trapezoid or second metacarpal fracture.^4,13,14,16-20,22^ In this subgroup, only 1 case of trapezoid stress fracture was reported;^4^ the remaining cases involved the second metacarpal, with the base and shaft almost equally affected (9 vs 8 cases respectively, Table 2). The dominant hand was injured in all patients, and they all complained of either wrist or hand pain with racket gripping, serving, or forehand stroking, similarly to our patient. On physical examination, point tenderness over the region of the second metacarpal and trapezoid was evident in multiple cases,^4,13,14,16-19,22^ and all patients except 2 had nonpainful full active ROM of the wrist and fingers. Of these 2 patients, 1 reported pain with index finger extension and the other reported mild pain at the extremes of the ROM.^4,19^ Swelling or induration was reported in 38% of cases (8 cases)^14,18,20^ and a hard mass in 1 case.^14^ These cases demonstrate the diversity in clinical presentation as described earlier.

**Table 2.** Reports of Nontraumatic Racket Sports–Related Stress Fractures of the Trapezoid and Second Metacarpal Bones

						Play Time		
Study	Age, years/Sex	Signs and Symptoms	Dominant Hand	Stress Fracture Location	Type of Sport	Hours Per Day	Increase in Training Intensity	Grip
Murakami, 1988^16^	16/M	Dorsal wrist pain specifically during serving and forehand stroke Point tenderness on base of 2nd metacarpal No swelling Nonpainful full active ROM of the wrist and fingers	Yes	Base of 2nd metacarpal	Tennis	2-3	No	NA
Waninger and Lombardo, 1995^17^	14/F	Hand pain with simple racket gripping Point tenderness over 2nd metacarpal No swelling Nonpainful resisted ROM of the wrist and fingers	Yes	Base of 2nd metacarpal	Tennis	NA	Yes	Western
Bespalchuk et al, 2004^18^	15/F	Hand pain with simple racket gripping and during stroking and serving Point tenderness over 2nd metacarpal Swelling of radial-dorsal aspect of the hand Nonpainful full active ROM of the wrist and fingers	Yes	Base and shaft of 2nd metacarpal	Tennis	3-5	Yes	Western
Muramatsu and Kuriyama, 2005^14^	13/F	Pain at dorsal aspect of 2nd metacarpal especially during stroking Point tenderness, swelling, and hard mass over 2nd metacarpal	Yes	Base of 2nd metacarpal	Soft tennis	3	Yes	Western
Fukuda et al, 2008^19^	14/F	Dorsal wrist pain especially with extension of index finger Point tenderness over base of 2nd metacarpal Nonpainful full active ROM of the wrist and finger except index finger extension	Yes	Base of 2nd metacarpal	Badminton	3-8	No	Western
Balius et al, 2010^20^	17/F	Mechanical pain at dorsum of hand and inability to wield a racket Induration at 2nd and 3rd metacarpal base (all patients) ROM: NA	Yes (all patients)	Shaft of 2nd metacarpal	Tennis (all patients)	NA	Yes	Western
	17/F			2nd metacarpal				Semi-western
	15/F			Shaft of 2nd metacarpal				Semi-western
	17/M			2nd, 3rd, 4th metacarpal				Eastern
	15/F			Shaft of 2nd metacarpal				Semi-western
	17/F			2nd metacarpal				Western
Blomquist et al, 2013^4^	21/F	Progressive wrist pain Point tenderness over the dorsal trapezoid with minimal tenderness over the scapholunate interval Full active ROM of the wrist mildly painful at extremes	Yes	Trapezoid	Tennis	NA	NA	NA
Duarte et al, 2017^13^	27/M	Hand pain especially when serving and forehand stroke Tenderness of the hand on palpation (exact location NA) ROM: NA	Yes	Shaft of 2nd metacarpal	Tennis	1	Yes	Eastern
Nishikawa et al, 2020^22^	13/F	Dorsal hand pain Point tenderness on dorsal aspect of the hand No swelling, ecchymosis, mass, or deformities Nonpainful full active ROM of the wrist and fingers	Yes	Shaft of 2nd metacarpal	Badminton	NA	NA	NA
	14/F			Base of 2nd metacarpal	Soft tennis			
	14/M			Base of 2nd metacarpal	Badminton			
	15/M			Base of 2nd metacarpal	Tennis			
	16/M			Shaft of 2nd metacarpal	Tennis			
	16/F			Base of 2nd metacarpal	Tennis			
	18/F			Shaft of 2nd metacarpal	Tennis			
	22/F			Shaft of 2nd metacarpal	Tennis			

F, female; M, male; NA, not available; ROM, range of motion.

Other factors that played an important role in these stress fractures were the duration and intensity of training and the athlete's grip technique. The cases reported in Table 2 show that most players either trained several hours daily^14,16,18,19^or had recently increased their training intensity,^13,14,17,18,20^ and they used the western/semi-western grip technique^14,17-20^ rather than the eastern grip technique.^13,20^ Balius et al mainly focused on the change in training intensity, especially in forehand strokes, which they believed to be a more important factor in stress fractures than grip technique.^20^ Nevertheless, other cases showed that the western grip played a more significant role than increased training intensity in such fractures.^14,17-19^

In tennis, the player's hand and wrist are subjected to immensely high pressure and force that are transmitted from the racket during every stroke.^13,16,17,20^ Repeated strokes with a poor grip technique cause repetitive microtrauma that can lead to second metacarpal and trapezoid bone fractures.^4,6,13,14,16,19,23^ As indicated by the cases reported in Table 2, the western grip technique seems to be associated with more injuries than the eastern grip technique. The main difference between the techniques is the position of the palm with respect to the racket handle (Figures 1 and 2). In the eastern grip, the palm is perpendicular to the racket surface, but the palm is parallel in the western grip, altering the biomechanics and increasing the weight on the second metacarpal, thus transferring all the tension to its base.^17,20^

The correlation between training intensity and technique explains why managing such injuries not only depends on rest and avoidance of sports but also on changing the grip technique or improving the biomechanics of the strokes to decrease the tension on the base of the second metacarpal.

## CONCLUSION

This case highlights the relation between trapezoid and second metacarpal stress fractures in athletes and shows the importance of history and physical examination in diagnosing such rare fractures, especially because of their varying clinical presentations. Trapezoid stress fractures should be considered in every differential diagnosis for athletes, especially for racket sport players presenting with wrist pain. Clinicians should identify the mechanism of the injury as soon as possible and be able to address not only the fracture itself but also the extent of play time, the modification of training dynamics and intensity, and the grip technique for all racket sport athletes to help prevent future injuries.

## Notes

*This article meets the Accreditation Council for Graduate Medical Education and the American Board of Medical Specialties Maintenance of Certification competencies for Patient Care and Medical Knowledge.*
